# Phage combination alleviates bacterial leaf blight of rice (*Oryza sativa* L.)

**DOI:** 10.3389/fpls.2023.1147351

**Published:** 2023-04-19

**Authors:** Hubiao Jiang, Changxin Li, Xuefang Huang, Temoor Ahmed, Solabomi Olaitan Ogunyemi, Shanhong Yu, Xiao Wang, Hayssam M. Ali, Fahad Khan, Chengqi Yan, Jianping Chen, Bin Li

**Affiliations:** ^1^ State Key Laboratory of Rice Biology and Breeding, Ministry of Agriculture Key Laboratory of Molecular Biology of Crop Pathogens and Insects, Key Laboratory of Biology of Crop Pathogens and Insects of Zhejiang Province, Institute of Biotechnology, Zhejiang University, Hangzhou, China; ^2^ Hunan Provincial Key Laboratory for Biology and Control of Plant Diseases and Insect Pests, Hunan Agricultural University, Changsha, China; ^3^ Taizhou Academy of Agricultural Sciences, Taizhou, China; ^4^ Ningbo Jiangbei District Agricultural Technology Extension Service Station, Ningbo, China; ^5^ Department of Botany and Microbiology, College of Science, King Saud University, Riyadh, Saudi Arabia; ^6^ Tasmanian Institute of Agriculture, University of Tasmania, Launceston, TAS, Australia; ^7^ Institute of Biotechnology, Ningbo Academy of Agricultural Sciences, Ningbo, China; ^8^ State Key Laboratory for Managing Biotic and Chemical Threats to the Quality and Safety of Agro-products, Key Laboratory of Biotechnology in Plant Protection of Ministry of Agriculture and Zhejiang Province, Institute of Plant Virology, Ningbo University, Ningbo, China

**Keywords:** phage, bacterial leaf blight, *Xanthomonas oryzae* pv. *oryzae*, phyllosphere microbiome, biocontrol mechanism

## Abstract

Rice bacterial leaf blight (BLB) is the most destructive bacterial diseases caused by *Xanthomonas oryzae* pv*. oryzae* (Xoo). Phages have been proposed as a green and efficient strategy to kill bacterial pathogens in crops, however, the mechanism of action of phages in the control of phyllosphere bacterial diseases remain unclear. Here, the glasshouse pot experiment results showed that phage combination could reduce the disease index by up to 64.3%. High-throughput sequencing technology was used to analyze the characteristics of phyllosphere microbiome changes and the results showed that phage combinations restored the impact of pathogen invasion on phyllosphere communities to a certain extent, and increased the diversity of bacterial communities. In addition, the phage combination reduced the relative abundance of epiphytic and endophytic Xoo by 58.9% and 33.9%, respectively. In particular, *Sphingomonas* and *Stenotrophomonas* were more abundant. According to structural equation modeling, phage combination directly and indirectly affected the disease index by affecting pathogen Xoo biomass and phage resistance. In summary, phage combination could better decrease the disease index. These findings provide new insights into phage biological control of phyllosphere bacterial diseases, theoretical data support, and new ideas for agricultural green prevention and control of phyllosphere diseases.

## Highlights

(1) Phages J2, J3 and E effectively reduce disease index of rice bacterial blight.
(2) Phage combination increases phyllosphere community diversity by reducing Xoo relative abundance.
(3) Rice bacterial blight index is associated with key phyllosphere ecological clusters.
(4) Phages J2, J3 and E reduce disease index by reducing Xoo biomass.
(5) Phage combination enhances Xoo resistance to phage and reduces its growth ability.

## Introduction

Rice (*Oryza sativa *L.) is an important cereal food crop, of which bacterial leaf blight (BLB) is one of the most devastating bacterial diseases, primarily caused by *Xanthomonas oryzae* pv. *oryzae* (Xoo) (Ahmed et al., 2022). It has the potential to reduce rice productivity while threatening food security (Mansfield et al., 2012; Liu et al., 2014). To control the outbreak of infection, several strategies have been employed, the most common of which is the use of resistant species, pesticides (copper-based chemicals), antibiotics, etc. (Peters et al., 2003; Niño-Liu et al., 2006; Zhang and Wang, 2013; Jambhulkar and Sharma, 2014; Van Hop et al., 2014; Kering et al., 2019). However, prolonged pesticide usage has caused toxic components to accumulate in the environment, development of copper-resistant pathogens, and it negatively impact human health through the food chain (Adrees et al., 2015; Costa et al., 2018; Lamichhane et al., 2018; Kering et al., 2019). Human medical conditions related to copper toxicity, include gastrointestinal, liver, and neurological illnesses (Alzheimer’s disease) (Roychoudhury et al., 2016; Navarro and Schneuwly, 2017; Kerkar and Roberts, 2018; Kering et al., 2019). Resistance to copper-based fungicides is also a challenge in controlling of the pathogen, and copper resistance has been reported in numerous plant diseases, including *Pseudomonas* and *Xanthomonas* spp. (Abbasi et al., 2015; Colombi et al., 2017; Richard et al., 2017; Kering et al., 2019). Notably, due to the widespread use of antibiotics in agriculture, resistance has been reported in the pathogens, such as streptomycin resistance in *Erwinia*, *Pseudomonas*, and *Xanthomonas* spp., and in plant pathogens. Horizontal gene transfer can contribute to the spread of antibiotic resistance *via* genes such as *strAB* (Lee et al., 2005b; Hyun et al., 2012; Xu et al., 2013; Sundin and Wang, 2018; Kering et al., 2019). Even though resistant types are considered to be the most effective and environmentally friendly for managing BLB (White and Yang, 2009; Fahad et al., 2014; Dong et al., 2018). However, the long development cycle of resistant cultivars, makes it hard and complex to establish resistance easily, hence, other alternative strategies for managing these pathogens need to be explored.

Phages are the most abundant biological organisms in the world; these are viruses that can replicate inside bacterial cells. The non-toxicity of phages to eukaryotic cells, self-replication, host specificity, ability to overcome antibiotic resistance, and ease of production make phages attractive as biocontrol agents. Food production has used phage therapy (Stone, 2002; Endersen et al., 2014), aquaculture (Nakai and Park, 2002), and even in the medical field (Kutter et al., 2010; Young and Gill, 2015). Also, phages are used to control plant bacterial diseases. For example, the utilization of mixed phages (including CHF1, CHF7, CHF19, and CHF21) effectively reduced the disease index of kiwifruit canker (Flores et al., 2020). Similarly, Wang et al. (2019) reported that phage combination reduced the incidence of tomato bacterial wilt up to 80%. Rabiey et al. (2020) demonstrated that employing phages significantly lowered the disease index of cherry by decreasing the number of *Pseudomonas syringae* pathogens. Additionally, there have been extensive research on the use of phages in the prevention and treatment of plant bacterial diseases. Phages are abundant in nature having specificity characteristics in targeting pathogenic bacteria without affecting the microbial community structure of the surrounding environment. These characteristics makes phages to be a potential agent for plant pathogen management.

Phages generally regulate pathogen growth through ecological and evolutionary mechanisms. The ecological mechanism regulates pathogenic bacteria density by improving the ecological niche of the native microbial community which gives rise to changes in the composition and diversity of surrounding microorganisms thereby increasing competition between pathogenic bacteria and native microbial communities while trying to wipe out pathogenic bacteria (Wang et al., 2019). However, the evolutionary mechanism is a trade-off between phage resistance evolution and pathogen resistance evolution. Phage-mediated strategy which decreases the density of pathogenic bacteria or evolutionary trade-offs may affect the diversity and function of microbial communities (Wang et al., 2019). Phages can substantially influence the structure of bacterial communities (Koskella and Brockhurst, 2014; Morella et al., 2018) and maintain bacterial diversity through a series of strategies (Korytowski and Smith, 2017; Maslov and Sneppen, 2017; Morella et al., 2018). The bacteriolytic effect of lytic phages on host bacteria regulates the amount and diversity of bacterial communities (Koskella and Brockhurst, 2014; Morella et al., 2018). Phages also enhance bacterial density and diversity by releasing nutrients into the environment through lysis (Weitz and Wilhelm, 2012; Morella et al., 2018). Thus, studies of phages in microbial communities can reveal interactions between bacteria and phages. Some studies proposed that lytic phages can considerably affect the relative abundance (RA) of dominant populations and intra- and inter-host diversity in the short term, indicating that lytic phages plays a significant role in phyllosphere microbial communities (Morella et al., 2018).

Although studies have reported in detail the interaction between phages and rhizosphere microbial communities. However, there is still a lack of evidence on what role phages play in phyllosphere microbial communities. This study aimed to investigate the effect of phage therapy on Xoo in the rice phyllosphere community and how it affects the phyllosphere microbial community. Our research findings will provide theoretical basis and technical support for agricultural green prevention and control of phyllosphere bacterial diseases.

## Materials and methods

### Pot experimental design

Glasshouse experiments were conducted using rice (Nipponbare) surface sterilisation seeds (75% ethanol for 2 minutes, 1% NaClO solution for 10 minutes). The rice seeds were washed three times with ddH_2_O and germinated for 48 hours on a sterile moistened triple-layer of filter paper. After germination, uniform seedlings were sown in plastic pots (14 cm diameter, 16 cm height) filled with (0.5 kg) sterilized peat moss which were placed in a greenhouse (light-dark photoperiod 14:10 h, temperature 24–30°C and 80% humidity). The experiment was laid out in a randomized block design and the seedlings were watered every two days using tap water. The experiment comprised seven groups: blank control (CK), pathogenic bacteria (C2), pathogenic bacteria and single phage (C2J2, C2J3, C2E), mixed phage (23E), and mixed phage and pathogenic bacteria (C223E), each treatment consisted of five replicates with each replicate consisting of 30 rice plants. The pathogen suspensions were diluted with sterile deionized water to a concentration of 10^7^ CFU×mL^−1^ cells. Pathogen Xoo:C2 srtain (Accn. no MH158532) was obtained from State Key Laboratory of Rice Biology and Breeding, Institute of Biotechnology, Zhejiang University, 310058 Hangzhou, China). Xoo (C2) was routinely grown at 30°C in nutrient broth (NB) medium (glucose 5.0 g×L^−1^, peptone 10.0 g×L^−1^, NaCl 10.0 g×L^−1^, beef extract 3.0 g×L^−1^) for 24 hours with shaking (170 rpm/min) before all the experiments.

### Pathogen inoculation and disease statistics

At the third-leaf stage, the Xoo pathogen suspension was evenly sprayed on rice leaves until the leaves were completely wet. After 24 hours the plants were inoculated with different phage combinations at a concentration of 10^6^ CFU×mL^−1^. A total of 30 rice plants were used with 2 mL of pathogenic bacteria suspension and 2 mL of phage suspension. Control plants were treated with an equal volume of NB liquid medium. At 15 days post foliar inoculation, the incidence statistics on rice plants were carried out according to the bacterial blight classification standard (in leaf units): grade 0: no disease, grade 1: spot area of less than 10% of leaf area, grade 3: spot area of 11% to 25% of leaf area, grade 5: spot area of 26% to 45% of leaf area, spot area of 46% to 65% of leaf area, spot area of leaf area of 65% or more. The calculation formula is as follows: incidence rate = spot area/leaf area. Disease index = ∑ (number of diseased leaves at each level × relative level)/(total number of surveyed leaves × highest level value) × 100%.

### Determination of resistance and growth capacity of pathogen Xoo

The resistance of evolved Xoo and ancient Xoo to initial inoculation phages was examined to understand better the evolutionary dynamics of Xoo phage resistance. The resistance of Xoo to phage was defined by the ratio of Xoo growth when inoculated with phage (OD_600+phage_) to Xoo growth alone (OD_600+CK_)(Wright et al., 2018). The specific process is as follows: 10 μL of Xoo (10^6^ CFU×mL^−1^) activated for one day and night was inoculated into 180 μL of NB medium, and 10 μL each of the three strains of single phage suspension (10^7^ pfu×mL^−1^) were added, with 10 μL NB medium serving as the control. After 24 hours of culture at 30°C, 170 rpm×min^−1^, the OD600 was measured. Resistance calculation for Xoo: resistance = OD_600+phage_/OD_600+CK_. The resistance of Xoo in which three phage combinations evolved to the initially inoculated phage was the average of the resistance of the three phages present in the pot experiment. Furthermore, the detailed procedures for measuring the maximum density of the evolved Xoo are as follows. Briefly, 10 μL of the activated Xoo was inoculated into 190 μL of NB liquid medium which was shaken at 170 rpm/min at 30°C for 48 hours. The OD_600_ was measured every 6 hours and the maximum biomass was estimated using the acquired growth curve, which was used to characterize the strain’s growth ability.

### Sample collection, DNA extraction, and sequencing

At 15 days post inoculation, rice leaf samples were collected for further analysis. From each treatment, 6–8 pieces of rice leaves were mixed in a 50 mL sterile centrifuge tube containing 40 mL of 0.01 M sterile PBS buffer (pH=7.4, 1 phosphate buffered saline liquid). At room temperature, ultrasonication at 40 kHz for 20 minutes was proceeded by shaking at 200 rpm×min^−1^ for 40 minutes. The leaves surface washing solution was passed through a 0.22 m sterile filter membrane, which was then cut into 22 mm squares for later usage. For phyllosphere endophytic microorganism investigation, collected rice leaves were surface-sterilized with 75% ethanol for 1 minute which was rinsed with sterile water three times. This was followed by the addition of 3% sodium hypochlorite to disinfect the leaves for 3 minutes and washed three times with sterile water. After snap freezing with liquid nitrogen and crushing to a powder with a sterile mortar and pestle, total genomic DNA samples were extracted using the OMEGA Soil DNA Kit (D5625-01) (OMEGA Bio-Tek, Norcross, GA, USA). The samples were then stored at −20°C until further analysis as specified by the manufacturer. The NanoDrop ND-1000 spectrophotometer (ThermoFisher Scientific, Waltham, MA, USA) and agarose gel electrophoresis were used to evaluate the quantity and quality of extracted DNA. **Table S1** shows the primers of epiphyllous and endophytic samples. Multiplex sequencing was carried out by incorporating 7 bp sample-specific barcodes into the primers. PCR products were purified with Vazyme VAHTSTM DNA Clean Beads (Vazyme, Nanjing, China China) and quantified using the Quant-iT PicoGreen dsDNA Assay Kit (Invitrogen, Carlsbad, CA, USA). Sequencing libraries were generated using TruSeq Nano DNA LT Library Prep Kit (Illumina, USA) following manufacturer’s recommendations. Afterward, the library quality was assessed on the Agilent Bioanalyzer 2100 system and 2 × 250 bp pair-end sequencing was accomplished through the Illumina MiSeq system (Shanghai Personal Biotechnology Co., Ltd., Shanghai, China).

### Bioinformatics analysis

All analyses of sequencing data were performed in QIIME2 software (Quantitative Insights Into Microbial Ecology 2 (Bolyen et al., 2019). First, primers were cut using the cutadapt plugin (Martin, 2011). The DADA2 plugins were quality filtered, denoised, and chimeras removed (Callahan et al., 2016). Taxonomy assignment to ASVs (amplicon sequence variants) was carried out using the classify learn naive Bayes classifier (Bokulich et al., 2018) based on the SILVA 132 database.

Sequence data analysis was mainly performed using QIIME2 and the R package (v3.6.0). Non-parametric statistical tests were performed (Kruskal-Wallis test) to assess alpha diversity and taxonomical differences at various stages. Based on Bray-Curtis distances, the beta diversity of microbial community structure was compared by principal coordinate analysis (PCoA), and PERMANOVA (Permutational multivariate analysis of variance, McArdle, and Anderson) were performed to assess the significant differences of microbial community structure between groups. The significant difference of clusters of different groups was compared using ASV-based. The R package “Hmisc” and “graph” was used to calculate the phyllosphere microbial community co-occurrence network for endophytes and epiphytes (RA> 0.05, significant correlation *P* < 0.05, Spearman coefficient N > 0.7). The spearman correlation matrix was calculated using packages “Hmisc” and “igraph”. To reduce false positive results, we adjusted all *P*-values for multiple correlations using Beniamini and Hochberg false discovery rate (FDR) (Benjamini and Hochberg, 1995). Robust correlations with the Spearman correlation coefficients > 0.70 and FDR-adjusted *P*-values < 0.01 were selected to construct the co-occurrence networks, which were visualized using the Fruchterman-Reingold layout with permutations in igraph. The relative abundance of the module was calculated by the “scale” function in R (Delgado-Baquerizo et al., 2018). In addition, the relationship between the ecological module and the disease index was tested by linear regression. The structural equation modeling (SEM) model was constructed using the robust maximum likelihood evaluation method in AMOS 26.0. Model fitness was evaluated using the chi-square, *P*-value, root mean square residual (RMR), root mean square error of approximation (RMSEA), and comparative fit index (CFI), goodness of fit index (GFI).

### Isolation and quantification of pathogen Xoo

A 1 g sample of rice leaves was surface sterilized with 75% ethanol for 1 minutes which was then rinsed three times with sterile water, followed by 3 minutes of surface disinfection with 1% sodium hypochlorite and three washes with sterile water. Thereafter, a sterile mortar was used to homogenize the leaf tissue. To identify the pathogen, leaf tissue suspensions were evenly applied onto the TSA medium and incubated in incubation boxes at 30°C for 3–5 days. Fifty Xoo strains were selected at random from each treatment sample, purified by scratching, and stored at −80°C. Furthermore, in order to count the Xoo in the tissue fluid, the classic bacterial counting method was used. Briefly, 100 μL of tissue suspension was added into 900 μL of sterile water and mixed well to make gradient dilution which was then smeared on the plate.

### Determination of the effects of phage combinations on phyllosphere bacterial communities

To obtain the natural bacterial community in the phyllosphere, the leaf surface washing solution and the leaf grinding solution were mixed with sterile water at 1:9 (w:v, g:mL). A 100 µL of leaf natural colonies was inoculated into 24-well plates containing 900 µL of NB medium. The experiment comprised of seven groups: blank control (CK), pathogen Xoo (C2, final concentration: 10^6^ CFU×mL^−1^), phage combinations (23E, final concentration: 10^7^ CFU×mL^−1^), and mixed phage and pathogenic bacteria (C223E). After shaking the culture at 30°C, 170 rpm×minutes^−1^ for 48h, the bacteria were collected by centrifugation for amplicon sequencing.

### Statistical analysis

All statistical analyzes were performed using R version 3.6.3 (http://www.r-project.org/). Differences in alpha diversity, disease index were compared using the nonparametric statistical test Kruskal-Wallis test. Differences in microbial community structure between groups were assessed using PERMANOVA (permutational multivariate analysis of variance). DESeq2 (Love et al., 2014) was used to identify differences in the abundance of different taxa between groups.

## Results

### Phage reduces disease index and pathogen density

**Figure 1:**
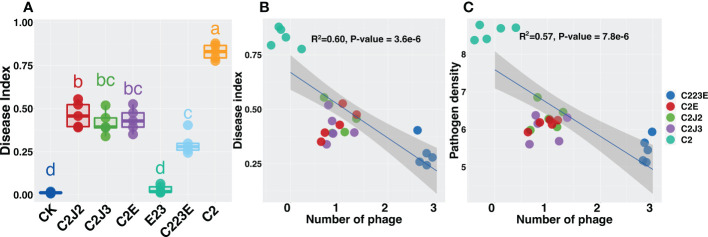
Effects of phages on rice bacterial blight disease index **(A, B)** and pathogen density **(C)**. All relationships were analyzed using linear regression analysis. R^2^ and *P*-value refer to the most parsimonious models. The different letter indicates a significant difference at the level of *P* < 0.05 by the Kruskal-Wallis test. CK: Control; C2: Xoo strain alone; C2J2, Xoo strain C2 + Xoo phage J2; C2J3, Xoo strain C2 + Xoo phage J3; C2E, Xoo strain C2 + Xoo phage E; C223E, Xoo strain C2 + Xoo phages (J2+J3+E).

As compared to the pathogen (C2), the phage treatment group considerably reduced the disease index of rice bacterial blight (**Figure 1A**). Phages reduced the disease index to varying degrees (44.0%–64.3%) and the disease index decreased as the number of phages increased (**Figure 1B**). The reduction in disease index was attributed to a decrease in the amount of pathogen (**Figure S1A**). Furthermore, as phage richness increased, the density of Xoo decreased significantly (**Figures 1C**, **S1B**). Notably, the number of phyllosphere pathogens decreased significantly after phage combination treatment (**Figure S1A**) and the decrease reached 100 times. Furthermore, significant differences were observed in treatment groups for single phage and phage combination. In summary, individual phages reduced the population of Xoo and the disease index of BLB while the phage combination significantly improved the control effect.

### Phages affect phyllosphere alpha diversity

**Figure 2:**
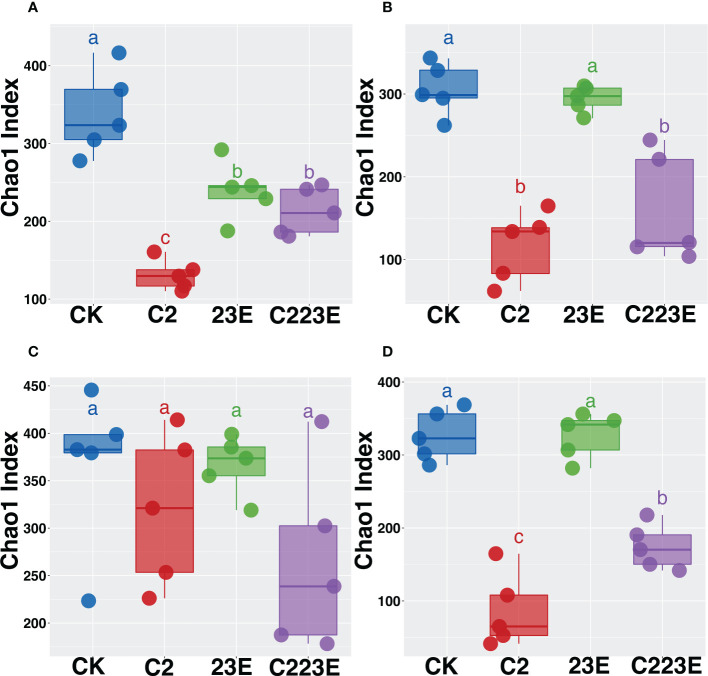
Effects of phage combination on the alpha diversity of epiphytic **(A, B)** and endophytic **(C, D)** bacterial communities. Alpha diversity is characterized based on the Chao1 index. Based on the Kruskal-Wallis test, different letters indicates significant differences at *P <* 0.05. CK, Control; C2, Xoo strain alone; 23E, Xoo phages (J2+J3+E); C223E, Xoo strain C2 + Xoo phages (J2+J3+E).

The alpha diversity index is a measure of species richness. The chao1 index of Xoo (C2) decreased significantly in the epiphytic microbial community 24 hours post inoculation when compared to the negative control (CK) (**Figure 2A**). Additionally, the chao1 index of phages and pathogenic bacteria (C223E) was higher than that of the C2 group (**Figure 2A**), indicating that phages ameliorated the decrease in the diversity of phyllosphere communities caused by pathogenic bacteria invasion and assisted the communities to recover to a certain extent. Furthermore, at 15 days post inoculation, there was no significant difference between the phage treatment group (23E) and the control group (CK) (**Figure 2B**), whereas the phage and Xoo (C223E) group were significantly higher than the Xoo (C2) (**Figure 2B**). Further analysis of the endophytic bacterial community in the phyllosphere revealed that there was no significant difference between all treatment groups 24 hours post inoculation (**Figure 2C**). The alpha diversity of the endophytic bacterial community of only Xoo (C2) group decreased significantly when compared to the control (CK) group (**Figure 2D**). Moreover, it was noted that the diversity of phages and Xoo (C223E) was significantly higher than that of the C2 group (**Figure 2D**). Furthermore, indoor culture experiments revealed that the pathogen Xoo significantly reduced the alpha diversity of the phyllosphere bacterial community while the introduction of phage combinations significantly relieved the diversity decrease caused by Xoo invasion and restored the community’s alpha diversity to its initial level (**Figures S6A, B**). Taken together, phage combinations indirectly affected the alpha diversity of phyllosphere communities by targeting Xoo.

### Phages affect microbial beta diversity in phyllosphere microbiota

**Figure 3:**
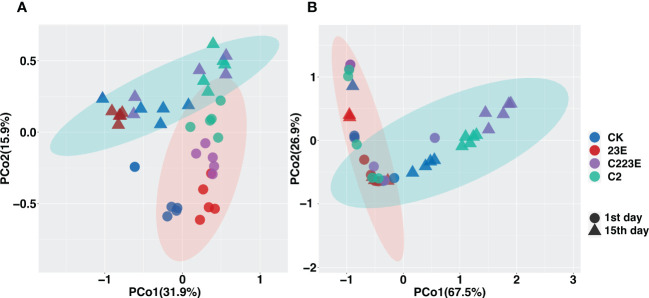
Distribution patterns of different treatment of phyllosphere bacteria-associated microbial communities. Principal coordinate analysis (PCoA) revealed the beta diversities of different treatment of phyllosphere epiphytes **(A)** and endophytes **(B)** bacterial communities. The black circle indicates the first day of inoculation and the black triangle indicates the 15th day post inoculation. CK, Control; C2, Xoo strain alone; 23E, Xoo phages (J2+J3+E); C223E, Xoo strain C2 + Xoo phages (J2+J3+E).

To analyze the epiphytic microbial community on the leaf, PCoA analysis was performed as shown in **Figure 3A**. The vertical axis explained 31.9% of the variation of the overall community while the horizontal axis explained 15.9% of the variation of the overall community (*P* = 0.001, **Figures 3A**; **S2A, B**). The bacterial communities on the phyllosphere at different sampling time points were observed to be separated along the horizontal axis, and different treatment groups were clustered together at different sampling time points. In addition, the permutational multivariate analysis of variance (PERMANOVA) was performed on all samples 24 hours post inoculation at two-time points of sampling. This was done to determine the disease and the effect size of different treatment groups. When all samples were analyzed together, BLB explained 10.9% of the community variation (*P* = 0.001) (**Table S2**), and different treatment groups explained 16.9% of the variation (*P* = 0.001) (**Table S2**). The samples were then separated based on sample time points for PCoA analysis (**Figure 3**). Considerable variation between the treatment groups 24 hours post inoculation was observed, explaining 36.1% (*P* = 0.001) (**Table S2**) while the different treatments explained 33.9% at 15th day post inoculation (*P* = 0.002) (**Table S2**).

In addition, we investigated the phyllosphere endophytic microbial community and it was noticed that as shown in **Figures 3B**; **S2C, D** the vertical axis explained 67.5% of the overall community while the horizontal axis explained 26.9% of the overall community (*P* = 0.001, **Figure 3B**). Furthermore, to determine the effect of size of BLB and different treatment groups we analyzed all samples at 24 hours post-inoculation at two sampling time points. First, it was discovered that BLB explained 10.9% of the community variation (*P* = 0.001) (**Table S2**) while different treatments explained 29.8% (*P* = 0.001) (**Table S2**). The samples were then separated based on the sampling time points for PCoA analysis (**Figure S2**). Significant variations between the treatment groups 24 hours post inoculation was observed, explaining 40.7% (*P* = 0.001) (**Table S2**). After the 15th day post-inoculation, different treatments explained 10.9% of the variance (*P* = 0.001) (**Table S2**). Notably, the results of indoor culture experiments showed that the pathogen Xoo invasion significantly altered the structure of the phyllosphere bacterial community (**Figures S6C, D** and **Table S2**). Conclusively, it was observed that different treatments significantly impacted the structure of the phyllosphere bacterial communities.

### Effect of phages on phyllosphere microbiota

**Figure 4:**
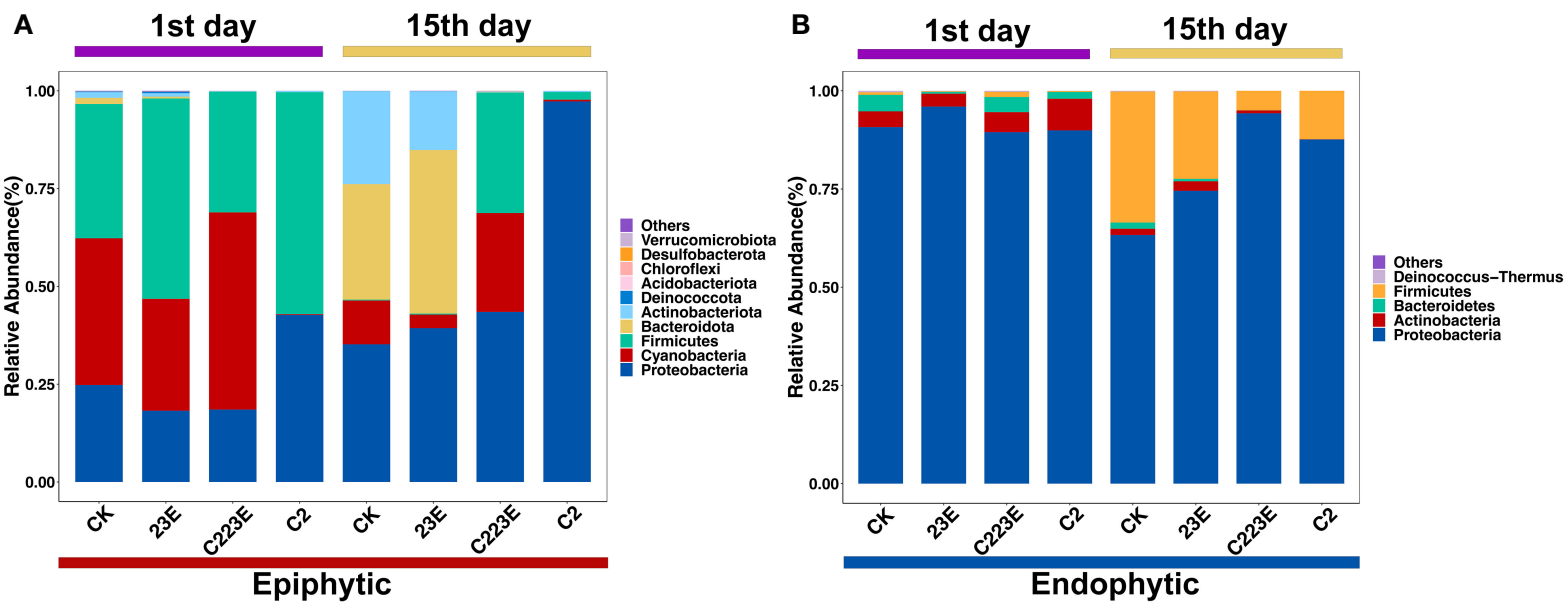
Taxonomic composition of the rice phyllosphere microbial community. RA of epiphytic **(A)** and endophytic **(B)** in different treatments. The phylum ranked outside the TOP10 were grouped into “Others”. CK, Control; C2, Xoo strain alone; 23E, Xoo phages (J2+J3+E); C223E, Xoo strain C2 + Xoo phages (J2+J3+E).

The bacterial composition of the phyllosphere in relation to various treatments revealed a considerable difference (**Figure 4**). At the phylum level, the bacterial communities in the epiphytic samples were represented by *Proteobacteria*, *Firmicutes*, *Cyanobacteria*, *Bacteroidota*, *Actinobacteriota*, and others. *Proteobacteria*, *Actinobacteria*, *Firmicutes*, *Bacteroidetes*, *Deinococcus Thermus*, as well as other endophytic samples were observed in the phyllosphere. Furthermore, the C223E treatment group significantly enriched 6 ASVs in the epiphyllous community, including *Sphingomonas*, *Exiguobacterium*, *Stenotrophomonas*, *Phyllobacterium*, and others when compared to C2 (**Table S3**). Although, the phyllosphere endophytes were not significantly enriched, they did depleted 8 ASVs. Notably, the RA of Xoo at the genus level decreased significantly by 88.7% 24 hours post inoculation in the epiphytic community (**Figure S3A**). However, at the 15th day post inoculation, the RA of Xoo in epiphytic and endophytic communities were decreased by 41.2 and 26.5%, respectively (*P* < 0.05) (**Figures S3B, C**). In addition, the RA of Xoo in the phyllosphere was negatively correlated with the disease index (**Figures S3D, E**) and the phage combinations significantly reduced the density of Xoo (**Figure S1**). According to these findings, phages significantly influence the species composition of epiphytic and endophytic bacterial communities by reducing the RA of Xoo.

### Co-occurrence networks of phyllosphere bacterial communities

**Figure 5:**
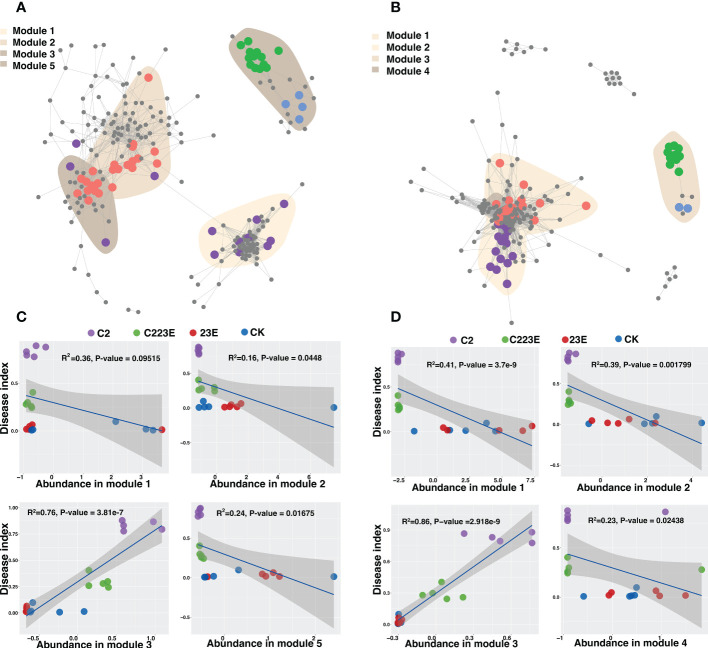
Co-occurrence patterns of phyllosphere bacterial communities. The co-occurrence network showed the connectivity between the core phyllosphere epiphytic ASVs **(A)** and phyllosphere endophytic ASVs **(B)**. (R > 0.7, *P* < 0.05; Indicated with gray lines). Gray nodes represents ASVs that were not sensitive to differences in different treatments; areas of different colors represents aggregation modules of phyllosphere dominant bacterial communities of different treatments; nodes of different colors corresponds to key dominant bacterial communities of different treatments. The lines between the nodes represents the correlation between the connected ASV. Regression of disease index and abundance of ecological modules in epiphytic **(C)** and endophytic **(D)** co-occurrence networks. CK, Control; C2, Xoo strain alone; 23E, Xoo phages (J2+J3+E); C223E, Xoo strain C2 + Xoo phages (J2+J3+E).

The symbiotic connections among ASV bacterial communities in the phyllosphere under different treatments were detected by co-occurrence network analysis. These responsive bacteria of epiphytic and leaf endophytic bacterial communities were focused primarily in various modules (**Figure 5**). The endophytic modules (Modules 1, 2, and 4) showed significant aggregation and overlap (**Figure 5B**) while the different epiphyllous community modules were separated (**Figure 5A**). In addition, different treatments also led to changes in the relative abundance of different ecological clusters in the co-occurrence network, and the relative abundance of module #3 in the epiphyllous and endophytic communities was significantly reduced (**Figures S4A, B**). Additionally, the relative abundance of module #2 and 5 in the phage-treated epiphytic communities was higher than in the control (**Figures S4A, B**). Module #3 in the epiphytic and endophytic communities was dominated by *Proteobacteria* (**Figures S4C, D**). Module #2 in the epiphytic community had a broad taxonomic range of bacteria (**Figure S4C**). Modules #1, 3, and 4 of the endophytic community were found to be mostly *Proteobacteria* while module #2 was somewhat *Firmicutes* (**Figure S4D**). Module #2 and #5, and the disease index in the epiphytic community were found to be significantly negatively correlated, while module #3 was positively correlated (**Figure 5C**). Only module#3 was found to be negatively associated with the disease index in the phyllosphere endophytic bacterial community whereas modules#1, 2, and 4 were all negatively correlated (**Figure 5D**).

### Ecological characteristics of phages biocontrol of BLB

**Figure 6:**
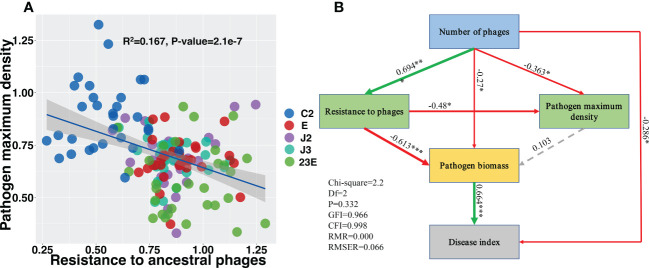
Evolved pathogen maximum density negatively correlated with resistance to ancestral phage **(A)**. Structural equation modeling showing the effects of phages, pathogen to phage resistance, and pathogen maximum biomass on the disease index **(B)**. Green lines indicates positive effects while red lines indicates negative effects. Dashed gray lines indicates insignificant effects. The width of the arrows indicates the strength of significant standardized path coefficients and the significance levels of each predictor are shown as *(*P* < 0.05), **(*P* < 0.01), and ***(*P* < 0.001). C2, Xoo strain alone; J2, Xoo phage J2; J3, Xoo phage J3; E, Xoo phage E; 23E, Xoo phages (J2+J3+E).

The resistance of Xoo in the offspring of the phage treatment group was significantly improved compared to Xoo (C2) group (**Figure S5A**) but there was no significant difference in Xoo resistance between the single phage treatment group and the phage combination treatment group (**Figure S5A**). Xoo’s ability to grow was further measured which was characterized by the maximum density. In comparison to the Xoo (C2) group, the growth ability of the phage treatment group’s offspring Xoo was significantly lowered (**Figure S5B**) and the growing ability of the phage combination was significantly reduced (**Figure S5B**). Furthermore, linear regression analysis revealed that Xoo’s resistance and growth ability were negatively correlated (**Figure 6A**, R^2^ = 0.167, *P* < 0.05). The sructural equation modelling determined the potential direct and indirect effects of phage number, pathogen resistance, pathogen growth ability (maximum density of Xoo), pathogen biomass, and rice bacterial blight disease index. The number of phages enhanced pathogen resistance to phages while lowering pathogen growth ability (**Figure 6B**). Bacteria phage resistance was negatively associated with bacteria biomass, while pathogens biomass was positively associated with disease index (**Figure 6B**). This implies that phage may indirectly minimize the amount of pathogen by mediating enhanced phage resistance in pathogenic bacteria and associated reductions in disease index. This suggests that phages could indirectly reduce the density of pathogen Xoo and the associated disease index by mediating the increase in pathogen resistance to phages.

## Discussion

The effects of single phage and combination of phages in the management of BLB and the ecological mechanism of phage biocontrol was evaluated in this study. The results showed that the phage combination treatment was better than single phage treatment in disease prevention and control. It is worth emphasizing that phages which are biological agents exert their control by directly lysing pathogenic bacteria. The mechanism of phage control is through ecological evolution, that is, the selection of pathogen Xoo with strong resistance to phages but weak growth ability. Additionally, through influencing pathogen Xoo, phages indirectly affect the composition and diversity of phyllosphere microorganisms and enrich potential beneficial bacteria in epiphytic community.

Studies have shown that phage combination can reduce a significant number of pathogenic bacteria than individual phages (Hall et al., 2012; Wei et al., 2017; Yu et al., 2018), which is consistent with the findings of this study (**Figures 1C**, **S1**). It was observed that all phages significantly reduced the BLB disease index with the phage combinations having the best control effect reducing it by 64.3% (**Figure 1A**). Additionally, it was observed that Xoo resistance was negatively connected to growth ability (**Figure 6A**). Structural equation modeling indicated that phage richness was positively correlated with Xoo resistance to ancestral phages but was negatively associated with the growth ability of pathogen Xoo (**Figure 6B**). These results strengthen prior research showing growing antibiotic resistance was caused mainly by the evolution of cross-resistant mutations (Betts et al., 2016; Betts et al., 2018; Wright et al., 2018).

The evolutionary cost of phage resistance is well known to be a cost reduction in pathogen-carrying capacity. Interactions between phages and bacteria have the potential to drive quick microbial evolution, and the antagonistic coevolution between the two is a major driver of microbial diversity, structure, and function (Scanlan, 2017). This is common in many phage-bacteria interactions, such as the interaction between *R. solanacearum* and its phages (Wang et al., 2017). All in all, the phage combinations play a major role in reducing the incidence of rice disease by selecting pathogenic bacteria with strong resistance to phage and limited their growth ability in the phyllosphere in order to maintain the density of the pathogen at a certain number while lowering the incidence of rice disease. Previous research has found that phage combinations can regulate the rhizosphere microbial community and composition, allowing the community to recover after *R. solanacearum* infection (Wang et al., 2019).

Our study found that phage combinations indirectly altered the diversity, community composition, and co-occurrence networks of phyllosphere microbial communities (**Figures 2**–**5**). Considering the high specificity of phages, this effect may be a phage-mediated effect that indirectly drives the reduction of the pathogen density in the phyllosphere and the reduction of the growth ability of the pathogen Xoo (**Figure 6B**). In addition, our laboratory experiments also verified this point (Wang et al., 2019), that is, phages can restore community diversity to an uninfected levels (**Figure S6**). This also shows that phage is an environmentally friendly and specific green biological resource for controlling pathogens. It is worth noting that, as compared to the Xoo (C2) treatment group, the RA of *Sphingomonas*, *Stenotrophomonas* and other beneficial bacteria increased significantly in the phage combination (C223E) treatment group (**Table S3**).

Among them, *Stenotrophomonas* is reported to be enriched in the rhizosphere of diseased wheat and can promote wheat growth and enhance wheat systemic resistance to improve plant disease resistance (Liu et al., 2021). In addition, *Stenotrophomonas maltophilia* suppresses rice blast, increases yield attributes and induces systemic resistance (Fariman et al., 2022). *Stenotrophomona rhizophila* increases the efficacy-biocontrol of *muskmelon rot* (Rivas-Garcia et al., 2019). Also, *Sphingomonas* was significantly enriched in the phyllosphere of citrus and it enhanced the inhibitory effect on the citrus pathogen *Diaporthe citri* by competing for iron (Li et al., 2022). In addition, *Sphingomonas* LK11 was previously reported to promote the growth of tomato plants during salt stress (Khan et al., 2014). It has been reported that *Sphingomonas melonis* can interfere with the sigma factor *RpoS* of the pathogenic plant *Burkholderia plantarii* by producing o-aminobenzoic acid, thereby blocking the synthesis of virulence factors, thereby enhancing the disease resistance of seeds (Matsumoto et al., 2021). As a consequence, we hypothesized that these beneficial bacteria may battle with pathogenic bacteria for nutrients in the phyllosphere, hence, affecting the occurrence of BLB. It was assumed that changes in the phyllosphere microbiome community driven by phages may increase the presence of antagonistic bacteria in the resident microorganisms. More functional analysis and culture experiments are needed to understand the function of enriched microorganisms in the future. Specifically, it is necessary to further isolate and cultivate the enriched potential beneficial bacteria, and test their disease-resistance and growth-promoting effects in order to draw more reliable conclusions.

Despite the reality that phages have mature applications in clinical, food health, and animal husbandry. However, because it has not been widely promoted in the field of plant disease prevention and control, its great potential in biological plant disease control has not been fully explored. This research reveals that the control effect of a phage combinations on rice bacterial blight is better than that of single phage, indicating that phage combinations can enhance biocontrol performance. At the same time, our research also provides possible solutions and data support for sustainable agricultural development and green disease prevention and biocontrol.

## Conclusions

Overall, our findings indicate that phages can effectively protect plants from Xoo infection. Meanwhile, the stability of the phyllosphere community was indirectly restored by targeting Xoo cleavage. Additionally, phage-induced increased pathogen resistance and reduced pathogen biomass played an important role in alleviating rice bacterial blight. Therefore, we provide technical support for field control of phyllosphere bacterial diseases, enrich biological control strategies, establish stable green bacterial disease control, and assist in the development of related application products.

## Data availability statement

The datasets presented in this study can be found in online repositories. The names of the repository/repositories and accession number(s) can be found below: NCBI accession PRJNA882604.

## Author contributions

BL and HJ conceived the idea and designed the experiment. CL, XH, SY, XW, HA, CY carried out the experiment. TA, HA, FK, HJ analyzed data and wrote the manuscript. SO, TA, BL, JC helped improve the quality of the manuscript. All authors contributed to the article and approved the submitted version.
