# Supplementary material for: Phage combination alleviates bacterial leaf blight of rice (Oryza sativa L.)
Source: Front Plant Sci. 2023 Apr 19;14:1147351. doi: 10.3389/fpls.2023.1147351 (PMC10155274; doi:10.3389/fpls.2023.1147351)
Supplement: Supplementary file 1 [file DataSheet_1.docx]

**Supplementary data**


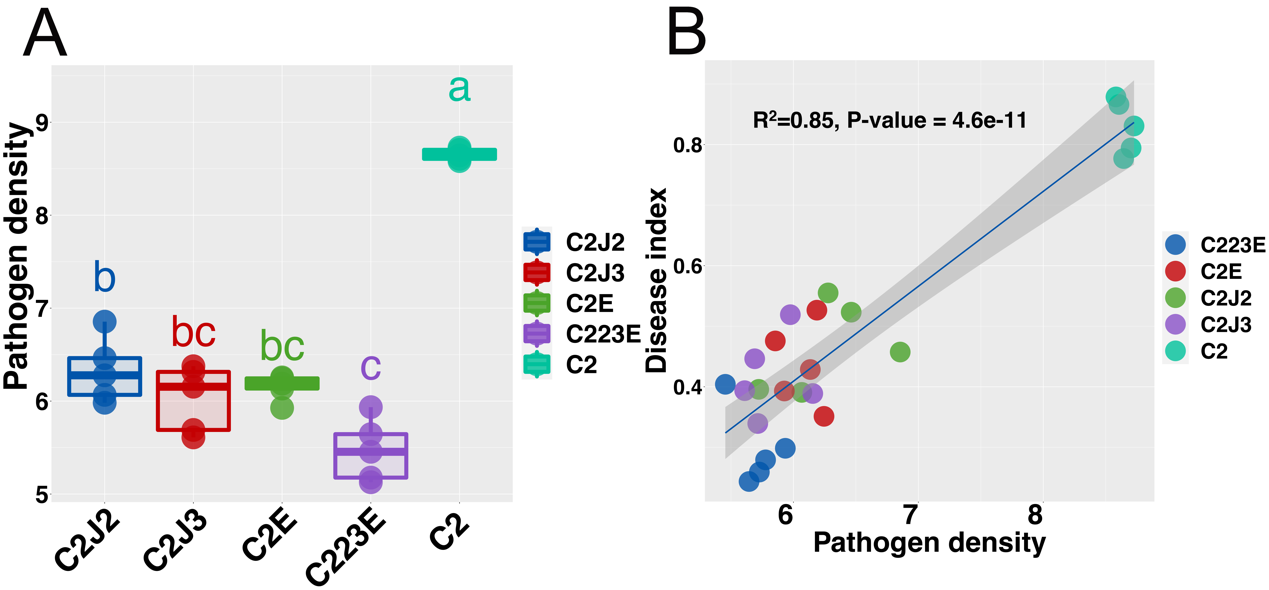


**Figure S1.** The effect of phage on the number of pathogenic bacteria (a) and the relationship between the density of pathogenic bacteria and the disease index (b). All relationships were analyzed using linear regression analysis. R^2^ and P-value refer to the most parsimonious models. The different letter indicates significant difference at the level of *P* < 0.05 by the Kruskal-Wallis test.


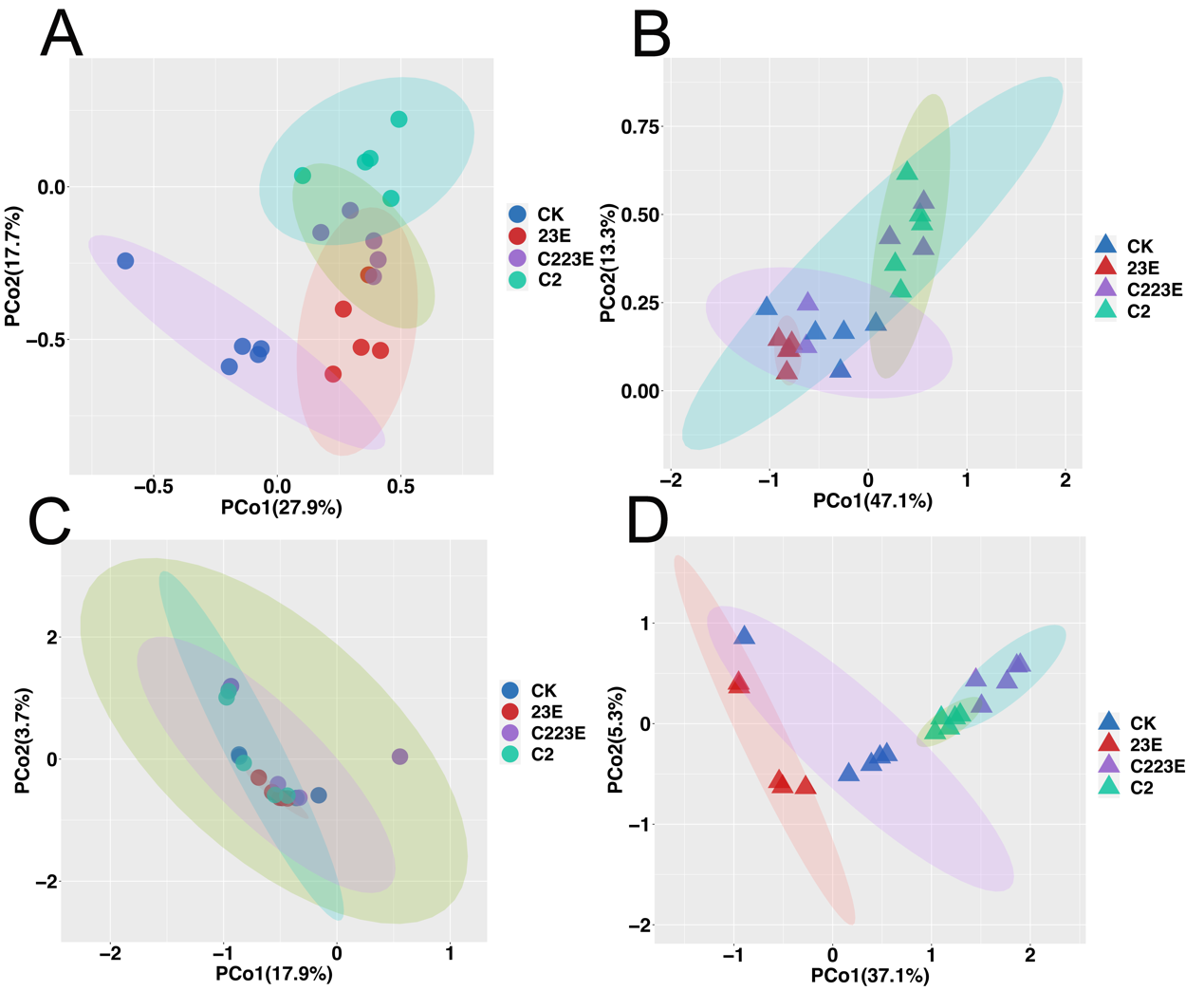


**Figure S2.** Distribution patterns of different treatment phyllosphere bacteria-associated microbial communities. Principal coordinate analysis (PCoA) revealed the beta diversities of different treatment phyllosphere epiphytic (a, b) and endophytic (c, d) bacterial communities.


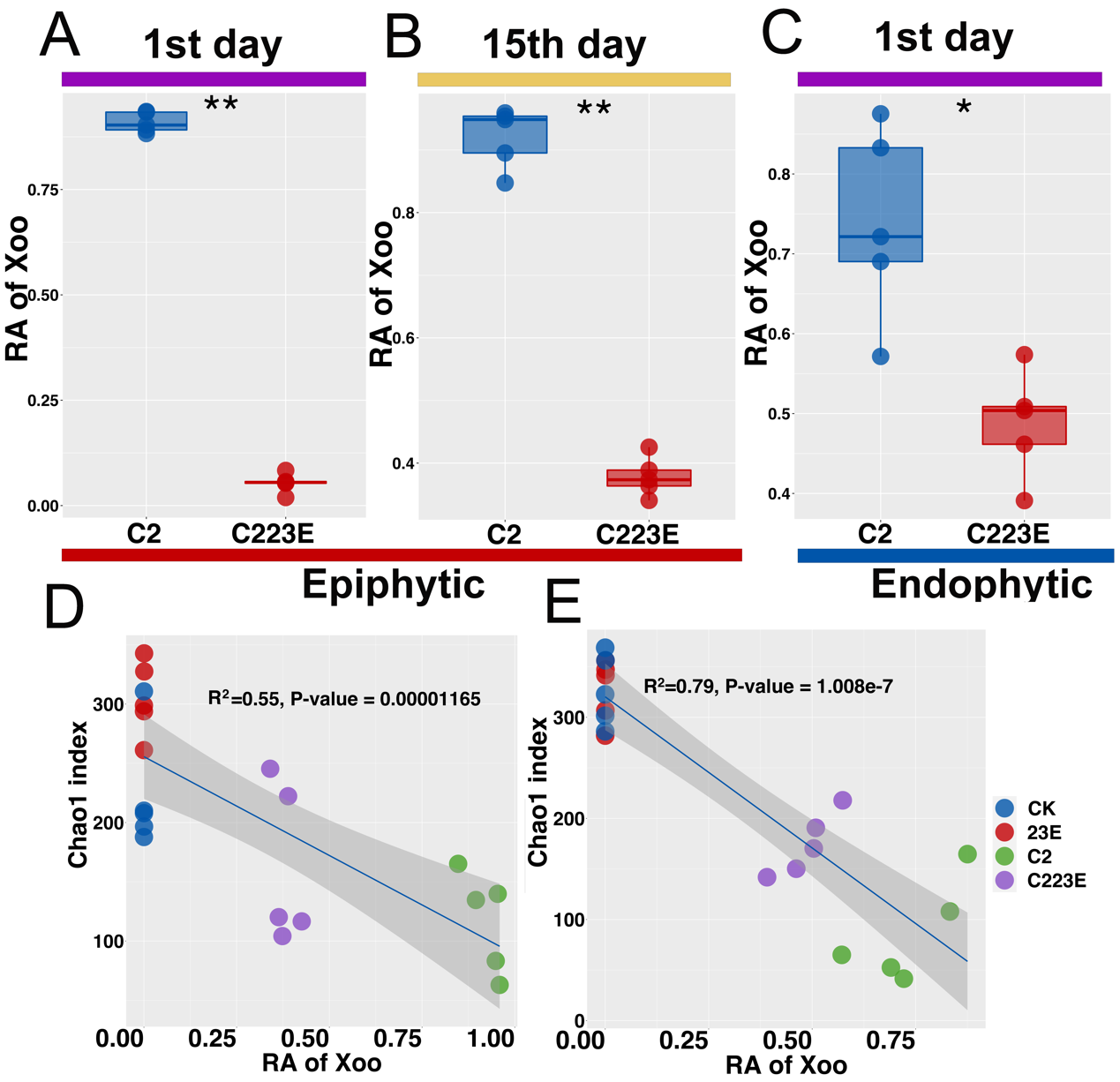


**Figure S3.** Relative abundance of the pathogen *Xoo* in epiphytic (a, b) and endophytic (c) bacterial communities. Linear regression analysis of *Xoo* relative abundance and chao1index in epiphytic (d) and endophytic (e) bacterial communities. Asterisks indicate significant differences (*: *P* < 0.05; **: *P* < 0.01). The significant difference was tested using the Wilcoxon rank-sum test.


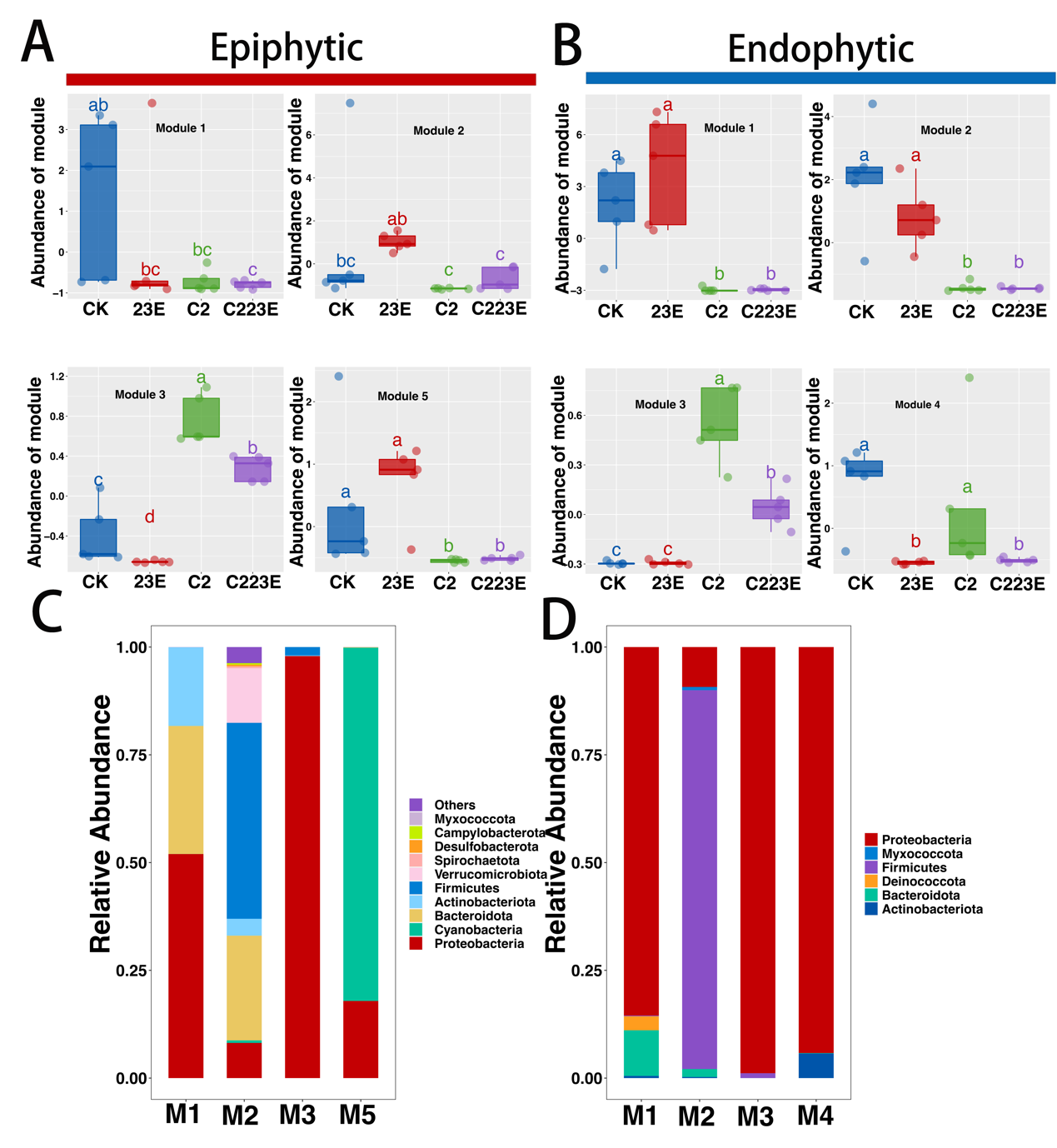


**Figure S4.** Relative abundance of ecological modules of epiphytic (a) and endophytic (b) bacterial communities under different treatments. Phylum-level taxonomic levels of sensitive ecological modules of epiphytic (c) and endophytic (d) bacterial communities. The different letter indicates significant difference at the level of *P* < 0.05 by the Kruskal-Wallis test.


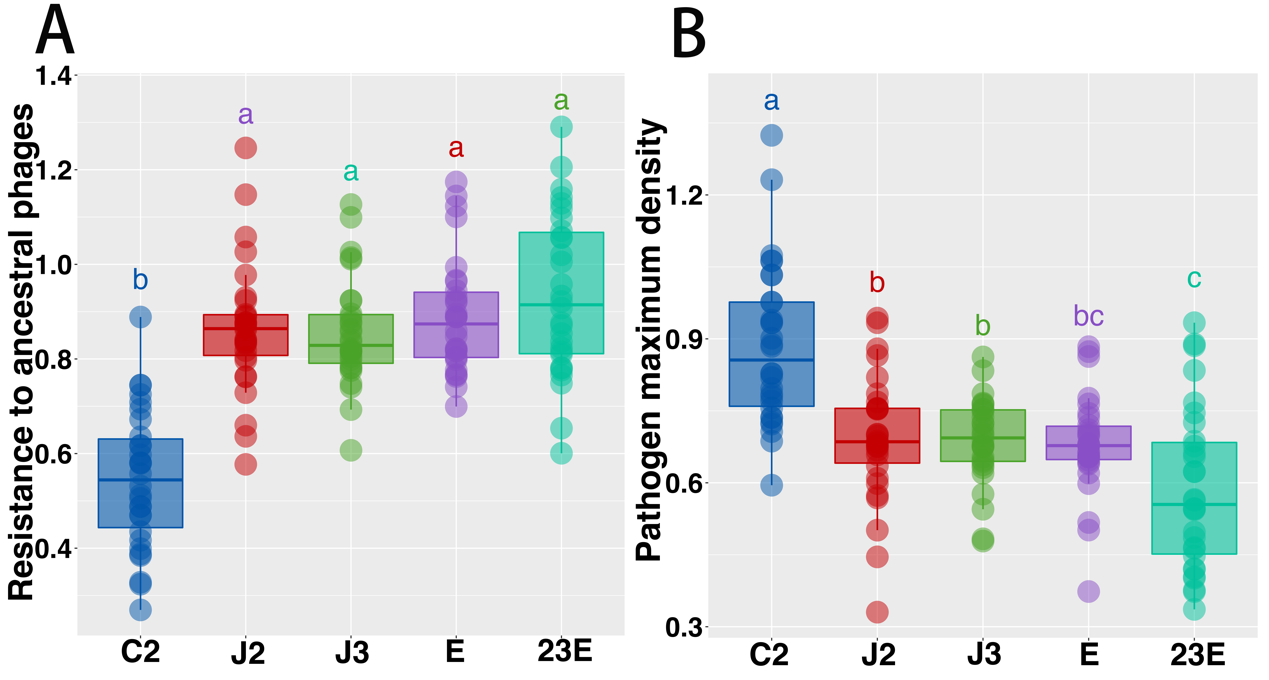


**Figure S5.** Evolved pathogen resistance to ancestral phages (a) and maximum biomass (b). Significant differences were determined using the Kruskal-Wallis test, where different lowercase letters indicate significant differences at *P* < 0.05.


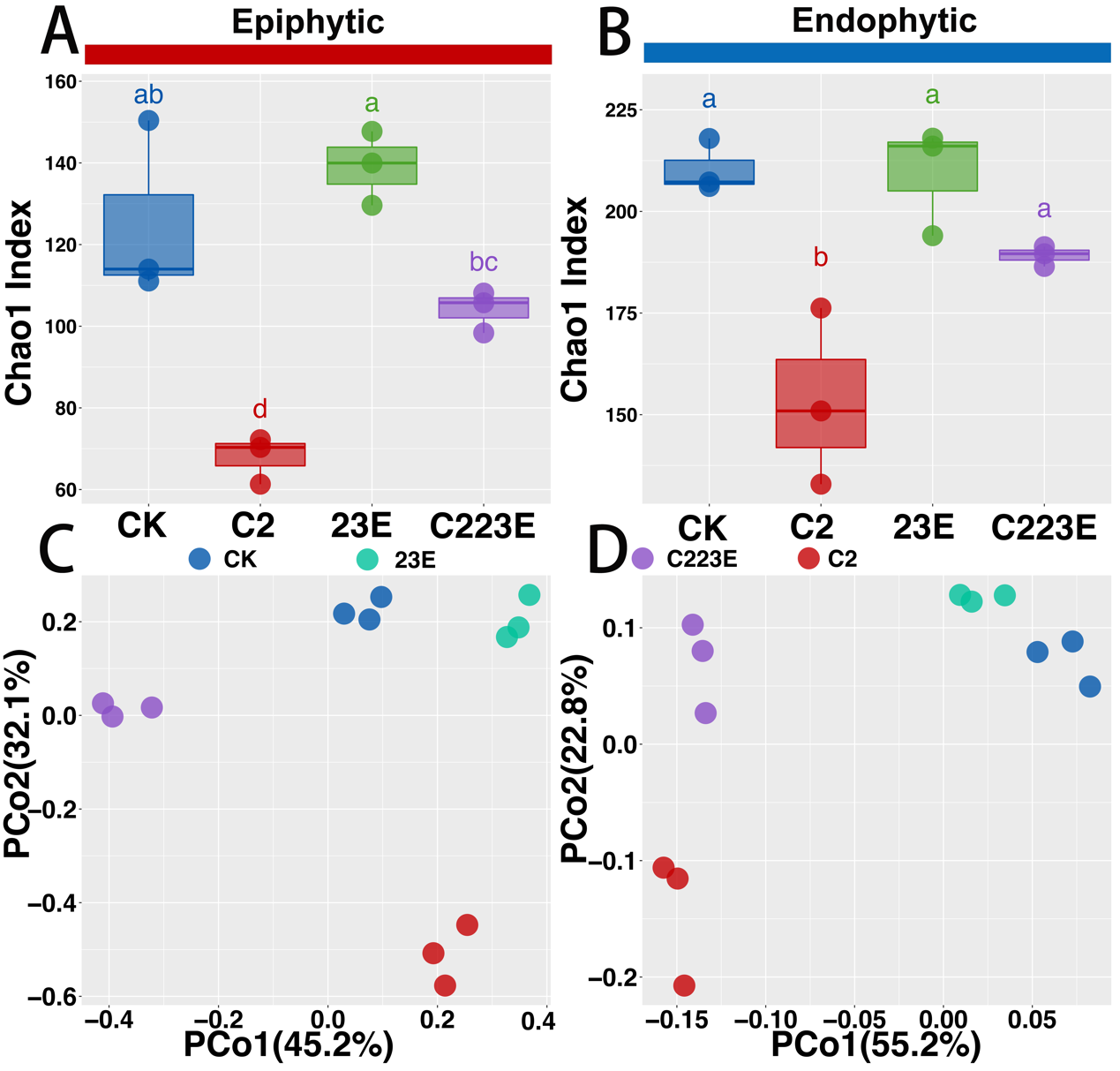


**Figure S6.** Effects of phage combinations on epiphytic and endophytic communities. ﻿ Effects of phage combination on the alpha diversity of epiphytic (a) and endophytic (b) bacterial communities. Alpha diversity is characterized based on the Chao1 index. Principal coordinate analysis (PCoA) revealed the beta diversities of different treatment phyllosphere epiphytic (c) and endophytic (d) bacterial communities. Different letters indicate significant differences at *P* < 0.05 based on Tukey’s test.

**Table S1. ﻿**Primers information used in this study

| **Gene fragments** | **Forward primer sequence** | **Reverse primer sequence** | **Gene description** |
| --- | --- | --- | --- |
| **16S rRNA** | 5’-CCTAYGGGRBGCASCAG-3’ | 5‘-GGACTACHVGGGTWTCTAAT-3’ | 341F and 806R |
| **16S rRNA** | 5‘-AACMGGATTAGATACCCKG-3’ | 5‘-ACGTCATCCCCACCTTCC-3’ | 799F and 1193R |
| **16S rRNA** | 5‘-AGAGTTTGATCCTGGCTCAG-3’ | 5‘-TACGGTTACCTTGTTACGACTT-3’ | 27F and 1492R |

**Table S2.** Significance test of beta diversity estimates between all samples.

| **Explanatory factors** | **R2** | **P** | **group** | **Method** |
| --- | --- | --- | --- | --- |
| Treatment | 0.169 | 0.001 | All epiphytic bacteria | PERMANOVA |
| Sampling time | 0.109 | 0.001 | All epiphytic bacteria | PERMANOVA |
| Treatment | 0.159 | 0.001 | All endophytic bacteria | PERMANOVA |
| Sampling time | 0.136 | 0.001 | All endophytic bacteria | PERMANOVA |
| Treatment | 0.361 | 0.001 | 1st day epiphytic bacteria | PERMANOVA |
| Treatment | 0.339 | 0.002 | 15th day epiphytic bacteria | PERMANOVA |
| Treatment | 0.03 | 0.09 | 1st day endophytic bacteria | PERMANOVA |
| Treatment | 0.239 | 0.002 | 15th day endophytic bacteria | PERMANOVA |
| Treatment | 0.983 | 0.001 | Indoor experiment of epiphytic community | PERMANOVA |
| Treatment | 0.962 | 0.001 | Indoor experiment of endophytic community | PERMANOVA |

**Table S3. ﻿**Differentially abundant analysis showing the enrichment and depletion patterns of phyllosphere bacterial taxa in C2 group compared C223E group.

| **Sample** | **ASVID** | **P-value** | **Level** | **Taxonomy** |
| --- | --- | --- | --- | --- |
| Epiphytic | ASV19 | 0.0002 | Enriched | Proteobacteria, Alphaproteobacteria, Sphingomonadales, Sphingomonadaceae, Sphingomonas |
| Epiphytic | ASV26 | 0.0110 | Enriched | Proteobacteria, Alphaproteobacteria, Rhizobiales, Rhizobiaceae, Phyllobacterium, Phyllobacterium myrsinacearum |
| Epiphytic | ASV38 | 0.0099 | Enriched | Proteobacteria, Alphaproteobacteria, Sphingomonadales, Sphingomonadaceae, Sphingomonas |
| Epiphytic | ASV27 | 0.0012 | Enriched | Firmicutes, Bacilli, Exiguobacterales, Exiguobacteraceae, Exiguobacterium |
| Epiphytic | ASV9 | 0.0093 | Enriched | Proteobacteria, Alphaproteobacteria, Sphingomonadales, Sphingomonadaceae, Sphingomonas |
| Epiphytic | ASV206 | 0.0114 | Enriched | Proteobacteria, Alphaproteobacteria, Rhizobiales, Beijerinckiaceae, Bosea |
| Epiphytic | ASV3 | 0.0215 | Enriched | Cyanobacteria, Cyanobacteriia, Chloroplast, norank, norank |
| Epiphytic | ASV44 | 0.0377 | Enriched | Proteobacteria, Gammaproteobacteria, Xanthomonadales, Xanthomonadaceae, Stenotrophomonas, Stenotrophomonas rhizophila |
| Epiphytic | ASV68 | 0.001 | Depleted | Proteobacteria, Alphaproteobacteria, Rhizobiales, Rhizobiaceae, Pseudochrobactrum |
| Epiphytic | ASV809 | 0.048 | Depleted | Proteobacteria, Alphaproteobacteria, Rhizobiales, Rhizobiaceae, Ochrobactrum, Ochrobactrum pseudogrignonense |
| Epiphytic | ASV25 | 0.046 | Depleted | Actinobacteriota, Actinobacteria, Corynebacteriales, Mycobacteriaceae, Mycobacterium |
| Epiphytic | ASV660 | 0.026 | Depleted | Proteobacteria, Gammaproteobacteria, Burkholderiales, Alcaligenaceae, Paenalcaligenes uncultured Alcaligenes sp. |
| Epiphytic | ASV161 | 0.001 | Depleted | Proteobacteria, Alphaproteobacteria, Rhizobiales, Rhizobiaceae, Paenochrobactrum |
| Epiphytic | ASV18 | 0.026 | Depleted | Proteobacteria, Gammaproteobacteria, Xanthomonadales, Rhodanobacteraceae, Rhodanobacter |
| Epiphytic | ASV73 | 0.045 | Depleted | Actinobacteriota, Actinobacteria, Corynebacteriales, Tsukamurellaceae, Tsukamurella |
| Epiphytic | ASV37 | 0.001 | Depleted | Proteobacteria, Gammaproteobacteria, Enterobacterales, Enterobacteriaceae, Enterobacter Enterobacter cloacae |
| Epiphytic | ASV81 | 0.000 | Depleted | Actinobacteriota, Actinobacteria, Micrococcales,Microbacteriaceae Leucobacter Leucobacter aridicollis |
| Epiphytic | ASV20 | 0.020 | Depleted | Actinobacteriota, Actinobacteria, Corynebacteriales, Mycobacteriaceae, Mycobacterium |
| Epiphytic | ASV88 | 0.000 | Depleted | Proteobacteria, Alphaproteobacteria, Caulobacterales, Caulobacteraceae, Brevundimonas uncultured bacterium |
| Epiphytic | ASV158 | 0.000 | Depleted | Proteobacteria, Gammaproteobacteria, Enterobacterales, Morganellaceae, Providencia Providencia rettgeri |
| Epiphytic | ASV30 | 0.009 | Depleted | Proteobacteria, Alphaproteobacteria, Rhizobiales, Beijerinckiaceae, Methylovirgula |
| Epiphytic | ASV40 | 0.000 | Depleted | Actinobacteriota, Actinobacteria, Micrococcales, Microbacteriaceae, Microbacterium |
| Epiphytic | ASV7 | 0.002 | Depleted | Actinobacteriota, Actinobacteria, Corynebacteriales, Tsukamurellaceae, Tsukamurella |
| Epiphytic | ASV11 | 0.002 | Depleted | Proteobacteria, Gammaproteobacteria, Burkholderiales, Burkholderiaceae, Burkholderia-Caballeronia-Paraburkholderia, Paraburkholderia fungorum |
| Epiphytic | ASV106 | 0.000 | Depleted | Firmicutes, Bacilli, Paenibacillales, Paenibacillaceae, Paenibacillus |
| Epiphytic | ASV4 | 0.000 | Depleted | Bacteroidota, Bacteroidia, Chitinophagales, Chitinophagaceae, Chitinophaga , uncultured Chitinophaga sp. |
| Epiphytic | ASV32 | 0.000 | Depleted | Firmicutes, Bacilli, Staphylococcales, Staphylococcaceae, Staphylococcus, Staphylococcus sciuri |
| Endophytic | OTU6 | 0.000 | Depleted | Proteobacteria, Gammaproteobacteria, Burkholderiales, Oxalobacteraceae, Herbaspirillum |
| Endophytic | OTU22 | 0.000 | Depleted | Proteobacteria, Gammaproteobacteria, Enterobacterales, Erwiniaceae, Pantoea, Pantoea agglomerans |
| Endophytic | OTU30 | 0.000 | Depleted | Proteobacteria, Gammaproteobacteria, Enterobacterales, Erwiniaceae, Pantoea |
| Endophytic | OTU32 | 0.000 | Depleted | Firmicutes, Bacilli, Bacillales, Bacillaceae, Bacillus |
| Endophytic | OTU140 | 0.000 | Depleted | Gammaproteobacteria, Burkholderiales, Burkholderiaceae, Burkholderia-Caballeronia-Paraburkholderia |
| Endophytic | OTU148 | 0.000 | Depleted | Bacilli, Bacillales, Bacillaceae, Bacillus, Bacillus aquimaris |
| Endophytic | OTU186 | 0.001 | Depleted | Firmicutes, Bacilli, Exiguobacterales, Exiguobacteraceae, Exiguobacterium |
| Endophytic | OTU76 | 0.005 | Depleted | Gammaproteobacteria, Xanthomonadales, Xanthomonadaceae, Stenotrophomonas, Stenotrophomonas maltophilia |
